# Comparison of PET imaging with a ^68^Ga-labelled PSMA ligand and ^18^F-choline-based PET/CT for the diagnosis of recurrent prostate cancer

**DOI:** 10.1007/s00259-013-2525-5

**Published:** 2013-09-27

**Authors:** Ali Afshar-Oromieh, Christian M. Zechmann, Anna Malcher, Matthias Eder, Michael Eisenhut, Heinz G. Linhart, Tim Holland-Letz, Boris A. Hadaschik, Frederik L. Giesel, Jürgen Debus, Uwe Haberkorn

**Affiliations:** 1 Department of Nuclear Medicine, University Hospital of Heidelberg, INF 400, 69120 Heidelberg, Germany; 2 Department of Radiopharmaceutical Chemistry, German Cancer Research Centre, Im Neuenheimer Feld 280, Heidelberg, Germany; 3 National Centre for Tumor Diseases (NCT)/DKFZ, INF 581, 69120 Heidelberg, Germany; 4 Department of Biostatistics, German Cancer Research Center, Im Neuenheimer Feld 280, Heidelberg, Germany; 5 Department of Urology, University Hospital of Heidelberg, Im Neuenheimer Feld 110, Heidelberg, Germany; 6 Department of Radiation Oncology, University Hospital of Heidelberg, INF 400, 69120 Heidelberg, Germany; 7 Clinical Cooperation Unit Nuclear Medicine, German Cancer Research Centre, Heidelberg, Germany

**Keywords:** Prostate cancer, PET/CT, Positron emission tomography, PSMA, Choline

## Abstract

**Purpose:**

Positron emission tomography (PET) with choline tracers has found widespread use for the diagnosis of prostate cancer (PC). However, choline metabolism is not increased in a considerable number of cases, whereas prostate-specific membrane antigen (PSMA) is overexpressed in most PCs. Therefore, a ^68^Ga-labelled PSMA ligand could be superior to choline tracers by obtaining a high contrast. The aim of this study was to compare such a novel tracer with standard choline-based PET/CT.

**Methods:**

Thirty-seven patients with biochemical relapse of PC [mean prostate-specific antigen (PSA) 11.1 ± 24.1 ng/ml, range 0.01–116] were retrospectively analysed after ^18^F-fluoromethylcholine and ^68^Ga-PSMA PET/CT within a time window of 30 days. Radiotracer uptake that was visually considered as PC was semi-quantitatively analysed by measuring the maximum standardized uptake values (SUV_max_) of the scans acquired 1 h after injection of ^68^Ga-PSMA complex solution (median 132 MBq, range 59–263 MBq) and ^18^F-fluoromethylcholine (median 237 MBq, range 114–374 MBq), respectively. In addition, tumour to background ratios were calculated.

**Results:**

A total of 78 lesions characteristic for PC were detected in 32 patients using ^68^Ga-PSMA PET/CT and 56 lesions were detected in 26 patients using choline PET/CT. The higher detection rate in ^68^Ga-PSMA PET/CT was statistically significant (*p* = 0.04). In five patients no lesion was found with both methods. All lesions detected by ^18^F-fluoromethylcholine PET/CT were also seen by ^68^Ga-PSMA PET/CT. In ^68^Ga-PSMA PET/CT SUV_max_ was clearly (>10 %) higher in 62 of 78 lesions (79.1 %) and the tumour to background ratio was clearly (>10 %) higher in 74 of 78 lesions (94.9 %) when compared to ^18^F-fluoromethylcholine PET/CT.

**Conclusion:**

^68^Ga-PSMA PET/CT can detect lesions characteristic for PC with improved contrast when compared to standard ^18^F-fluoromethylcholine PET/CT, especially at low PSA levels.

**Electronic supplementary material:**

The online version of this article (doi:10.1007/s00259-013-2525-5) contains supplementary material, which is available to authorized users.

## Introduction

Prostate cancer (PC) is the second most frequent cancer and the sixth leading cause of cancer death in men worldwide [1]. One of the key issues of this tumour entity is to detect recurrent disease. To date this is a major challenge for all conventional imaging modalities [2]. Although choline-based positron emission tomography (PET)/CT is widely used for this purpose, there have been numerous studies reporting a low sensitivity and specificity, especially at low prostate-specific antigen (PSA) levels [3–13]. Consequently, improved imaging of PC is necessary. One novel promising method is PET imaging with ^18^F-FACBC, a new synthetic amino acid. Recent evaluations by Nanni et al. indicate that this tracer might be superior when compared to choline PET/CT [14].

In addition, prostate-specific membrane antigen (PSMA) recently has received increased attention [15–19]. This cell surface protein is significantly overexpressed in PC cells when compared to other PSMA-expressing tissues such as kidney, proximal small intestine or salivary glands [20, 21]. It therefore provides a promising target for PC-specific imaging and therapy [22].

Recently methods have been developed to label PSMA ligands with ^68^Ga, ^99m^Tc and radioiodine enabling their use for PET or single photon emission computed tomography (SPECT) imaging and therapy [15, 16, 22–24]. Our initial experience with PET/CT using Glu-NH-CO-NH-Lys-(Ahx)-[^68^Ga(HBED-CC)] (^68^Ga-PSMA) as a ^68^Ga-labelled PSMA ligand suggests that this novel tracer can detect PC relapses and metastases with high contrast by binding to the extracellular domain of PSMA, followed by internalization [25, 26]. The aim of our study was to compare this novel tracer with standard choline-based PET/CT in the diagnostics of patients presenting a biochemical recurrence of the disease.

## Materials and methods

### Patient characteristics

For this study we originally selected 38 male patients who underwent both choline-based PET/CT and ^68^Ga-PSMA PET/CT within a time window of 30 days. One of the patients was deleted from the study as the histological evaluation revealed multiple metastatic and converging lymph node packages. This precludes radiological differentiation of individual lymph nodes and a meaningful quantitative comparison of lymph node detection rate between choline and PSMA PET/CT. Therefore, the total number of patients analysed in this study was 37. In all cases there was suspected progressive disease following prior conventional treatment of PC (e.g. hormone therapy, chemotherapy, radiation therapy and/or surgery).

All patients signed a written informed consent form for the purpose of anonymized evaluation and publication of their data. The quantity of patients presented in this study reflects our data collected during the past 3 years. Follow-up could be conducted in 33 of 37 patients up until the time this manuscript was submitted.

With the exception of patients 26 and 29 (organizational reasons), all patients were first investigated by ^18^F-fluoromethylcholine PET/CT. This is currently the method of choice in routine clinical practice. However, due to unsatisfying imaging results of choline PET/CT and to evaluate possible treatment with ^131^I-labelled PSMA ligands (therapy data not yet published), further investigation using ^68^Ga-PSMA PET/CT was suggested. This additional PET scan was conducted only in case both patients and their referring physicians gave their consent.

All reported investigations were conducted in accordance with the Helsinki Declaration and with our national regulations. This study was approved by the Ethics Committee of the University of Heidelberg (permit S-321/2012).

Patient characteristics are summarized in Table 1. Twenty-eight patients had previously undergone prostatectomy (Table 1, patients 1, 3–4, 7–8, 10–16, 19, 22–23 and 25–37), whereas nine were treated with prior radiation therapy and androgen deprivation without surgical removal of the prostate. The average age was 69.3 ± 7.1 years (range 57–85, median 70.0) with a mean Gleason score (GSC) of 7.4 ± 1.1 (range 5–9, median 7.0) and a mean PSA level of 11.1 ± 24.1 ng/ml (range 0.01–116, median 4.0 ng/ml). PSA was measured in blood samples taken at the time of the first PET/CT scan. The average time between both investigations was 12.1 ± 8.4 days (range 1–30, median 11.0).

**Table 1:**
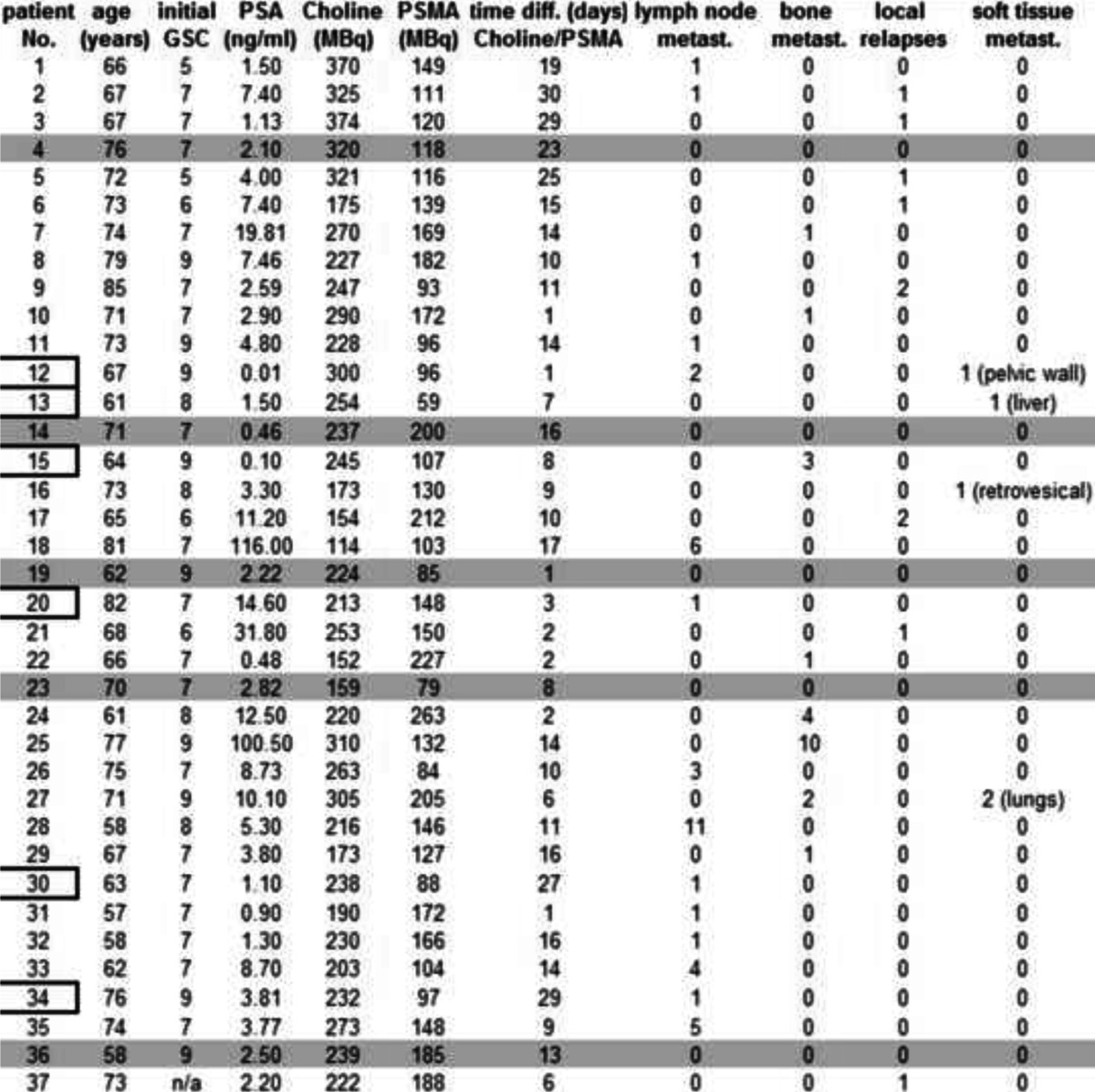
Patient characteristics

Grey fields: patients without pathological tracer uptake in both PSMA-ligand and Choline based PET/CT

Framed numbers: patients without pathological findings in Choline-PET/CT only

Fifteen patients (3, 5–14, 16–17 and 19–20) of the present study have coincidentally been analysed in a different study evaluating the biodistribution of the PSMA tracer in humans [26]. This concordance was not intentional. The authors are convinced that these patients had to be integrated into the present manuscript instead of being excluded from the novel study. Their inclusion improves data quality and confirms the findings of this study.

### Imaging

^68^Ga-PSMA PET/CT was obtained with the ^68^Ga-labelled HBED-CC conjugate of the PSMA-specific pharmacophore Glu-NH-CO-NH-Lys that was synthesized as described previously [16]. ^68^Ga^3+^ was obtained from a ^68^Ge/^68^Ga radionuclide generator and complexed with the HBED-CC conjugate as previously published [16, 17]. The final product was formulated in isotonic phosphate-buffered saline (PBS) with subsequent sterile filtration. The radiolabelling and purification of the PSMA ligand was done using an automated module. As a consequence, the radiochemical yield was not determined regularly. Typically, the labelling efficiency (radiochemical yield) is >98 % as determined by module validation. The ^68^Ga-PSMA complex solution was applied to patients via an intravenous bolus (mean 139.6 ± 46.3 MBq, range 59–263 MBq, median 132 MBq). Targeted ^68^Ga-PSMA was 2 MBq/kg. Variation of injected radiotracer activity was caused by the short half-life of ^68^Ga and variable elution efficiencies obtained during the lifetime of the ^68^Ge/^68^Ga radionuclide generator. However, in our experience with PSMA PET/CT during the last 3 years, all injected activities were sufficient in detecting PC (see also in the “Discussion”). All injections contained 2 nmol PSMA ligand resulting in a median specific radioactivity of 66 GBq/μmol.

Choline-based PET/CT was performed with ^18^F-labelled fluoromethylcholine (IASOcholine®, Iason, Graz, Austria). The production was according to common radiopharmaceutical standards and regulations. The choline solution was applied to patients via an intravenous bolus (mean 241.6 ± 59.4 MBq, range 114–374 MBq, median 237 MBq). Targeted choline activity was 3 MBq/kg.

A non-contrast-enhanced CT scan was performed 1 h post tracer injection using the following parameters: slice thickness of 5 mm, increment of 0.8 mm, soft tissue reconstruction kernel, 130 keV and 80 mAs. Immediately after CT scanning, a whole-body PET was acquired in 3D (matrix 164 × 164). For each bed position (16.2 cm, overlapping scale 4.2 cm) we used 4-min acquisition time with a 15.5-cm field of view (FOV). The emission data were corrected for randoms, scatter and decay. Reconstruction was conducted with an ordered subset expectation maximization (OSEM) algorithm with 2 iterations/8 subsets and Gauss-filtered to a transaxial resolution of 5 mm at full-width at half-maximum (FWHM). Attenuation correction was performed using the low-dose non-enhanced CT data. PET and CT were performed using the same protocol for every patient on a Biograph 6 PET/CT scanner (Siemens, Erlangen, Germany).

### Image analysis

Image analysis was performed using an appropriate workstation and software (Syngo TrueD, Siemens, Erlangen, Germany). Two board-certified specialists in nuclear medicine with 8 and 9 years of clinical experiences read all data sets independently and resolved any disagreements by consensus. Choline and PSMA PET/CT were analysed in a randomized fashion. Lesions that were visually considered as suggestive of PC were counted and analysed with respect to their localization (local relapses, lymph node, bone and soft tissue metastases) and to their maximum standardized uptake values (SUV_max_) as it is common practice in daily routine. SUV_max_ was chosen due to its higher reproducibility between different investigators when compared to SUV_mean_. The latter one is always dependent on the volume of interest (VOI) drawn by the investigator, whereas SUV_max_ is independent [26].

For calculation of the SUV, circular regions of interest were drawn around areas with focally increased uptake in transaxial slices and automatically adapted to a three-dimensional volume of interest at a 70 % isocontour. In the case of suspicious focal tracer uptake in one investigation and simultaneous virtually inconspicuous finding in the same area using the corresponding different PET/CT technique, SUV was measured in the inconspicuous areas as well.

SUV_max_ of the same lesions in both PSMA ligand- and fluoromethylcholine-based PET/CT were defined as clearly less, equal or clearly more with intensity differences of ≤10 %, between −10 and +10 % or >10 %, respectively. SUV values of the same lesions in both PSMA ligand- and fluoromethylcholine-based PET/CT as well as their ratio to background signal (in SUV_max_) were statistically analysed using a Wilcoxon signed rank test.

When analysing the contrast of lesions with pathological tracer uptake and therefore visually highly suggestive of PC (characteristic for PC), several background tissues corresponding to the localization of the lesions were selected. This method is more accurate and better reflects the contrasting ability of the imaging modality in the region of interest when compared to the selection of one general background tissue that might show differing background uptake than the region of interest. However, particularly in cases of a variety of metastases the selection of multiple backgrounds is not always feasible. For our study we chose the background according to the following algorithm:

In cases of bone metastases in the vertebral column, of local relapses, of lung metastases and of liver metastases, we selected adjacent normal tissue as background. In cases of non-vertebral bone metastases, we selected the contralateral normal bone tissue. In cases of soft tissue metastases and of lymph node metastases, the gluteal musculature was selected as background.

The selection of two different backgrounds in cases of bone metastases (vertebral and non-vertebral) as mentioned above is due to the well-known fact that the background signal in choline-based PET imaging is higher in the vertebral column when compared to other skeletal structures (e.g. Fig. 4d). The statistical differences between the background signal of the vertebral column and other parts of the skeletal system in choline PET/CT are described in the “Results”.

### Statistical analysis

For statistical analysis, Excel 2010 (Microsoft, Redmond, WA, USA) and SigmaPlot version 11 software (Systat Software, Inc., Chicago, IL, USA) were used. Significance of differences was evaluated by:

1. Two-sided Wilcoxon signed rank tests for tumour uptake and contrast in both PET/CT methods.
2. Two-sided paired *t* tests to evaluate differences concerning the background signal between choline- and PSMA-based PET/CT.
3. Two-sided unpaired two-sample *t* tests to evaluate differences concerning GSC and applied radioactivity between groups with and without pathological uptakes.
4. Two-sided Mann–Whitney tests to evaluate differences concerning PSA values between groups with and without pathological uptakes.
5. Two-sided McNemar test to analyse whether ^68^Ga-PSMA PET/CT detects significantly more lesions characteristic for PC when compared to choline-based PET/CT.

In all cases a *p* value of <0.05 was considered statistically significant. Furthermore, regression analysis between PSA and SUV_max_ was done for both investigations.

## Results

There were no adverse or clinically detectable pharmacological effects in any of the patients after injection of both tracers. In 32 of 37 (86.5 %) patients at least 1 lesion characteristic for PC was detected in ^68^Ga-PSMA PET/CT. By contrast, only 26 of 37 (70.3 %) patients presented with pathological findings in ^18^F-fluoromethylcholine PET/CT.

Using ^68^Ga-PSMA PET/CT 78 lesions characteristic for PC were detected in 32 patients and using ^18^F-fluoromethylcholine PET/CT 56 lesions were detected in 26 patients. The higher detection rate in ^68^Ga-PSMA PET/CT was significant (McNemar test, *p* = 0.04). In five patients no lesion was found with both methods (Table 1).

With PSA values of 2.82 ng/ml and less, at least one lesion characteristic for PC was identified in 68.8 % of patients, while all patients presented with pathological lesions at PSA values greater than 2.82 ng/ml. Using the same threshold for ^18^F-fluoromethylcholine PET/CT, 43.8 % of patients presented with at least one lesion at PSA levels of 2.82 ng/ml and less. In addition, 90.5 % of the patients presented with lesions at levels greater than 2.82 ng/ml. All lesions detected by ^18^F-fluoromethylcholine PET/CT were also seen in ^68^Ga-PSMA PET/CT.

Amongst all 78 lesions characteristic for PC, 40 were defined as lymph node metastases, 23 as bone metastases, 10 as local relapses and 5 as soft tissue metastases (1 of the pelvic wall, 1 hepatic, 1 retrovesical and 2 pulmonary PC metastases, all 5 confirmed by histology).

**Fig. 1:**
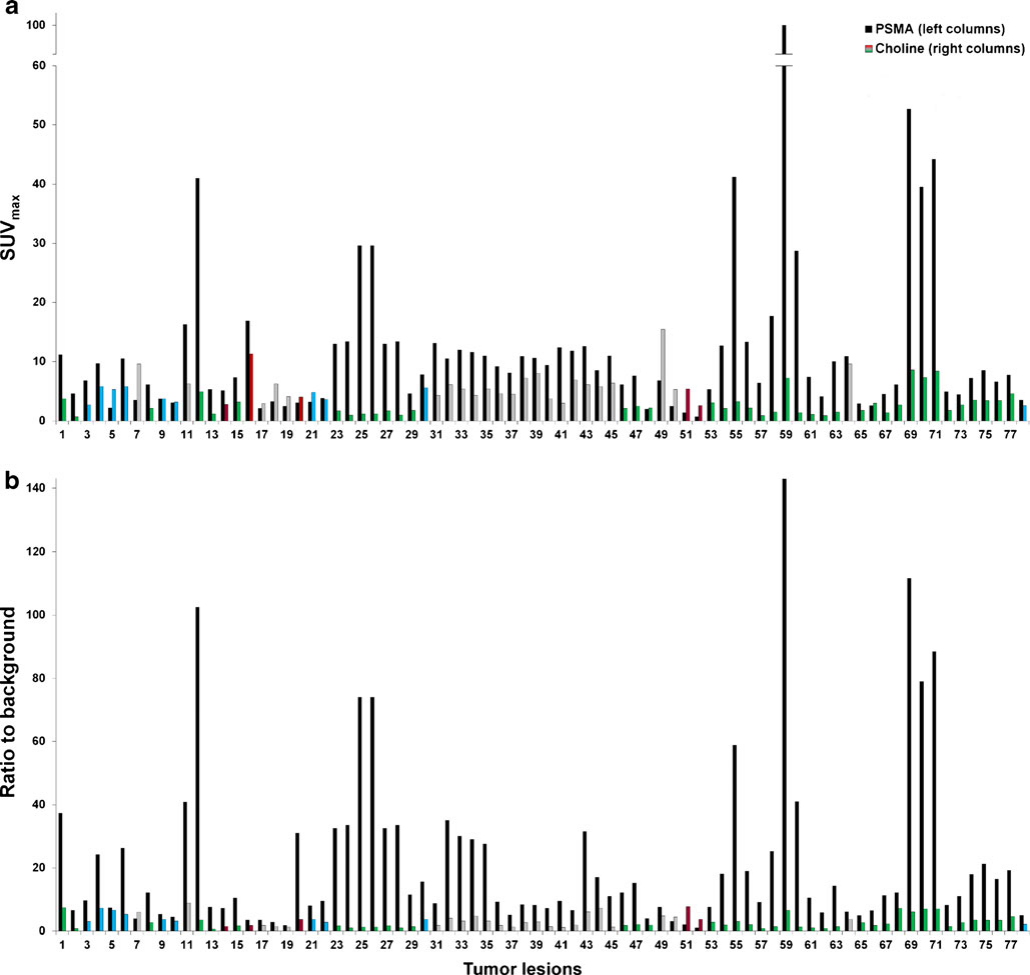
SUV_max_ (**a**) of all 78 lesions characteristic for PC and their ratio to background SUV_max_ (= contrast, **b**) 1 h post-injection in both ^68^Ga-PSMA PET/CT (*left columns*) and ^18^F-fluoromethylcholine PET/CT (*right columns*). *Green columns* indicate lymph node metastases, *blue columns* local relapses, *grey columns* bone metastases and *red columns* soft tissue metastases

Figure 1a demonstrates the SUV_max_ values of all 78 lesions and their origin. In ^68^Ga-PSMA PET/CT SUV_max_ was clearly (>10 %) higher in 62 of 78 lesions (=79.1 %, which was significant, *p* < 0.001), clearly (>10 %) lower in 12 lesions (15.4 %) and was equal to ^18^F-fluoromethylcholine PET/CT in 4 lesions (5.5 %).

Figure 1b demonstrates the tumour to background ratio which was clearly (>10 %) higher in 74 of 78 lesions (=94.9 %, which was significant, *p* < 0.001) when using ^68^Ga-PSMA. The four remaining lesions in two different patients presented with higher ratios in ^18^F-fluoromethylcholine PET/CT (Table 1, patients 7 and 27, Fig. 1, lesions 7 and 50–52).

Concerning both SUV_max_ of lesions and their signal to background ratio, the most significant differences between the two imaging methods were observed in lymph node metastases followed by bone metastases, local relapses and soft tissue metastases as demonstrated by Fig. 1 and Tables 2–3 in supplementary data.

**Fig. 2:**
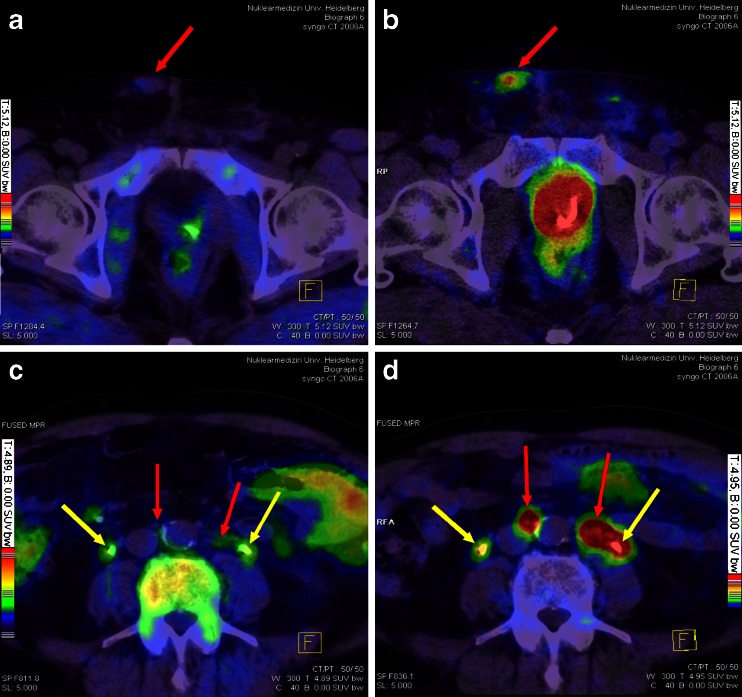
Patient 12 (**a**, **b**) and patient 18 (**c**, **d**). *Red arrows* point to a nodular pelvic wall metastasis (**a**, **b**, histologically confirmed) and to small lymph nodes (**c**, **d**) which present with clearly pathological tracer uptake in ^68^Ga-PSMA PET/CT (**b** and **d**) only. *Yellow arrows* point to both catheterized ureters (**c**, **d**). Patient 12 presented with a minimal PSA value (0.01 ng/ml) despite visible tumour lesions. The PSMA ligand is therefore able to detect low differentiated PC. **a** + **c** Fusion of ^18^F-fluoromethylcholine PET and CT, **b** + **d** fusion of ^68^Ga-PSMA PET and CT. Colour scales as automatically produced by the PET/CT machine

**Fig. 3:**
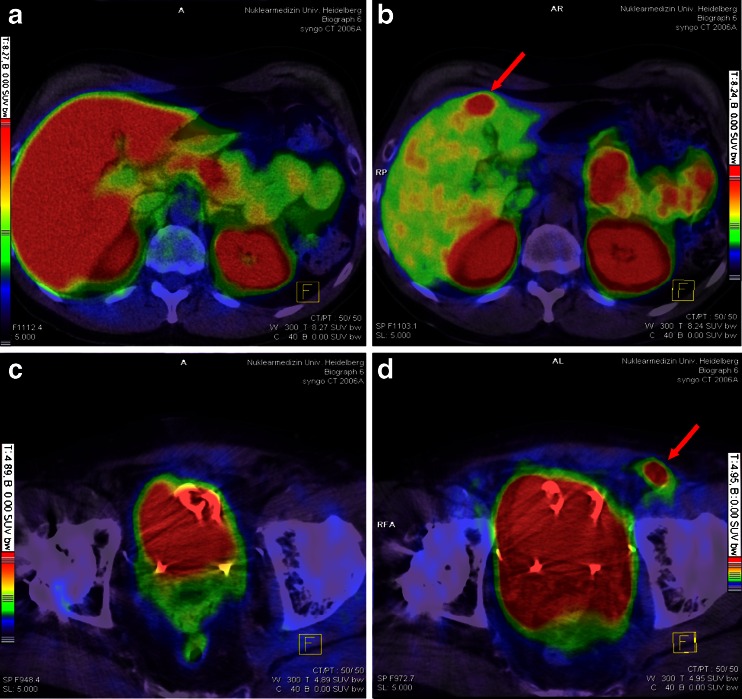
Patient 13 (**a**, **b**) and patient 18 (**c**, **d**). *Red arrow* in **b** points to a liver metastasis (histologically confirmed, lesion 16 in Fig. 1) visible only in ^68^Ga-PSMA PET/CT due to relatively low background activity when compared to ^18^F-fluoromethylcholine PET. In **d**, *red arrow* points to a lymph node which presents with clearly pathological tracer uptake in ^68^Ga-PSMA PET/CT despite a beam hardening artefact (lesion 28 in Fig. 1). In ^18^F-fluoromethylcholine PET/CT, however, there is no pathological uptake (**c**). **a** + **c** Fusion of ^18^F-fluoromethylcholine PET and CT, **b** + **d** fusion of ^68^Ga-PSMA PET and CT. Colour scales as automatically produced by the PET/CT machine

**Fig. 4:**
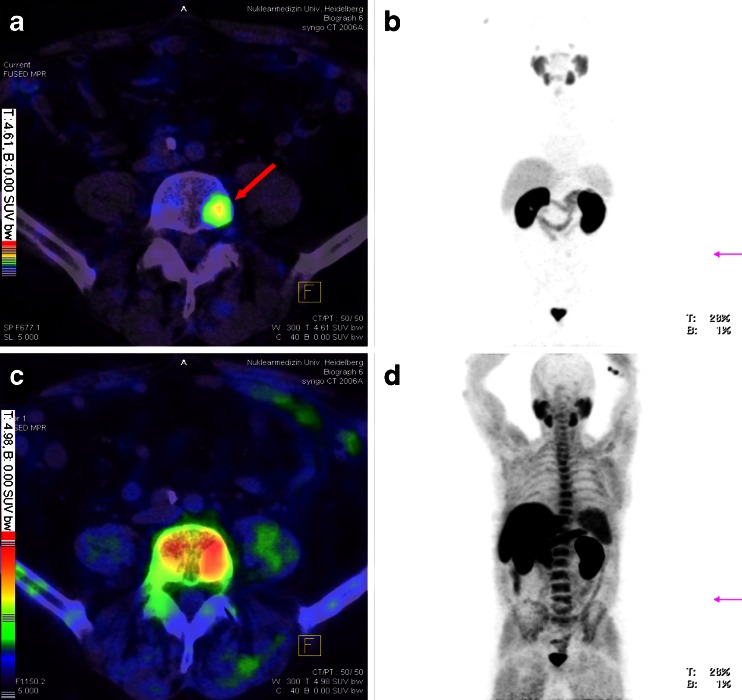
Patient 15. *Red arrow* points to a vertebral metastasis visible in ^68^Ga-PSMA PET/CT (**a**) only. Due to physiological high background activity in the vertebral column, vertebral metastases are usually difficult to detect in choline PET (**c**). Typical for choline PET is also a frequently high background activity as visible in the maximum intensity projection (MIP, **d**). Image data are given as automatically produced by the PET/CT machine to demonstrate that the filtering of the MIPs was equally set. Although it appears that the automatically set scales may not be optimal to detect lesions on the MIP in every case (e.g. lesion in **b** is better visualized on the workstation than in this figure), we preferred to avoid changes of all figures presented in this study. **a** Fusion of ^68^Ga-PSMA PET and CT, **b** MIP of ^68^Ga-PSMA PET, **c** fusion of ^18^F-fluoromethylcholine PET and CT, **d** MIP of ^18^F-fluoromethylcholine PET. Colour scales as automatically produced by the PET/CT machine

Figures 2, 3 and 4 demonstrate selected examples of the improved contrast when using the PSMA ligand.

In all selected background tissues, tracer uptake (as measured with SUV_max_) was significantly lower in ^68^Ga-PSMA PET/CT than in ^18^F-fluoromethylcholine PET/CT (two-sided paired *t* tests): *p* < 0.001 in gluteal musculature, *p* < 0.001 in vertebral bones, *p* < 0.001 in non-vertebral bones, *p* = 0.014 in lungs and *p* < 0.001 in the liver.

Specific characteristics of patients with and without pathological findings in ^18^F-fluoromethylcholine PET/CT are detailed in Table 4 (supplementary data). Between these two groups, the mean PSA level was significantly lower (two-sided Mann–Whitney test, *p* = 0.009) and GSC significantly higher in the group without pathological findings (*t* test, *p* = 0.038). The injected dosage was not significantly higher in the group with pathological findings (*t* test *p* = 0.87).

Specific characteristics of patients with and without pathological findings in ^68^Ga-PSMA PET/CT are also detailed in Table 4 (supplementary data). Between these two groups, injected dosage (*t* test, *p* = 0.76) and PSA level (two-sided Mann–Whitney test, *p* = 0.096) were not significantly lower in the group without pathological findings. The GSC was not significantly higher in the group without pathological findings (*t* test, *p* = 0.42). There was no relation in the regression analysis between PSA and SUV values in both choline- and PSMA-based PET/CT (raw data not shown).

Seven patients with pathological radiotracer uptake in ^68^Ga-PSMA PET/CT were further investigated by biopsy or surgery (Table 1, patients 11–13, 16–17, 27 and 32). In all cases PC was confirmed. No false-positive or false-negative lesions were found in all of these cases.

In addition, ten patients were treated by selected radiation therapy (patients 1, 3, 4, 6, 10, 21, 29–31 and 34). In all cases, PSA decreased significantly after radiation. One patient (35) was treated by radiation as well, but the first PSA evaluation after treatment was still pending at the time of manuscript submission.

Three patients (18, 25 and 33) were treated with ^131^I-labelled PSMA ligands. After treatment, PSA decreased significantly as well. Like selective radiation, these therapies demonstrated also that the PSMA-positive lesions were metastases of PC. Eight patients (7, 8, 20, 23, 24, 26, 28 and 36) were treated with androgen deprivation therapy (ADT) only. In all cases, PSA decreased as well. Four patients were referred for active surveillance (2, 5, 9 and 19) and four other patients were not available for follow-up (14, 15, 22 and 37).

Further explanation of patients 4 and 36: after initial PET/CT without pathological findings (part of this study), both patients were referred for a second PSMA PET/CT 1 year after the first investigation. In the case of patient 4, one nodular PSMA-positive lesion in the pelvis was found. This lesion was retrospectively already existent in the first PET/CT although it was smaller and did not present with pathological tracer uptake. In the case of patient 36, multiple lymph node metastases were found in the second scan only. Therefore, the first scan was obviously false-negative in both cases.

In summary, PSMA-positive lesions were proven to be PC by histology in seven cases. In 13 patients, selective radiation therapy and therapy with ^131^I-labelled PSMA ligands followed by a significant decrease of PSA were indicative of the fact that PSMA-positive lesions in PET/CT were metastases of PC.

## Discussion

To date, the detection of lesions in the context of biochemical recurrence of PC is a major challenge for all imaging modalities including choline-based PET/CT [2–13]. The aim of this study was to compare the established ^18^F-fluoromethylcholine PET/CT with a novel ^68^Ga-labelled PSMA ligand. We retrospectively analysed 37 patients who underwent both ^18^F-fluoromethylcholine PET/CT and ^68^Ga-PSMA PET/CT analysis within a time window of 30 days. In all cases there was suspected progressive disease following prior conventional treatment of PC. Most patients were initially referred for ^18^F-fluoromethylcholine PET/CT. However, due to reasons mentioned before, further ^68^Ga-PSMA PET/CT was conducted. Theoretically the order of investigations could introduce a bias due to possible tumour progression between the first and the second PET/CT. In reality however it is well known that PC usually presents with slow growth and noticeable changes within 30 days are very unlikely. Furthermore, the average time between both examinations was 11.9 days only.

In the following, numerous essential results of this study are highlighted and discussed in detail to further demonstrate the superiority of ^68^Ga-PSMA PET/CT when compared to choline. In 86.5 % of the patients at least one lesion characteristic for PC was detected in ^68^Ga-PSMA PET/CT. This rate is similar to our previous data [26]. By contrast, only 70.3 % of the patients presented with pathological findings in ^18^F-fluoromethylcholine PET/CT which is similar to the data reported in the literature [3–13]. ^18^F-Fluoromethylcholine PET/CT did not reveal any suspicious lesions in 11 patients, while only 5 patients presented without any pathological findings in ^68^Ga-PSMA PET/CT.

Especially at lower PSA levels, ^68^Ga-PSMA PET/CT detected more PC lesions when compared to choline. This was statistically significant as demonstrated in the results. These data are also similar to our previous report concerning this PSMA ligand [26]. Also the rates of choline are in agreement with the data reported in the literature [3–13]. Therefore, ^68^Ga-PSMA PET/CT proved to be clearly superior in detecting PC lesions at low PSA levels when compared to choline-based PET/CT.

A significant advantage of ^68^Ga-PSMA PET/CT is that lesions characteristic for lymph node metastases frequently presented with very high contrast when compared to choline (Fig. 1 and Table 2). ^18^F-Fluoromethylcholine PET demonstrated low sensitivity in detecting lymph node metastases. This disadvantage of choline has also been reported in the literature [6, 7]. The superior contrast in ^68^Ga-PSMA PET/CT has also been demonstrated in most skeletal metastases and local relapses (Fig. 1, Tables 2 and 3 in supplementary data). This is even more significant than the differences of uptake values in both methods and illustrates one main drawback of choline-based PET: even at acceptable uptake values, a high background signal frequently hampers the detection of lesions.

Our study, therefore, demonstrates that metastases and recurrent PC usually present with high contrast in ^68^Ga-PSMA PET/CT contributing to a significantly improved detection of PC lesions even at low PSA levels when compared to ^18^F-fluoromethylcholine PET/CT. We hypothesize that detection rates in ^68^Ga-PSMA PET/CT will increase with rising PSA levels and tumour size. Recently, Chondrogiannis et al. reported about a proportional correlation between trigger PSA and detection rate when using choline PET/CT [27]. On the other hand, there was no relation between PSA and SUV values in both choline- and PSMA-based PET/CT in our study. With regard to choline, our results are in agreement with the literature [28, 29]. For the PSMA tracer, however, no comparable data are available.

Only four PC lesions in two patients presented with a better contrast in ^18^F-fluoromethylcholine PET/CT. Possible explanations for this finding are low injected activity of the PSMA ligand, previous therapies and different biological properties such as PSMA expression, choline transport and phosphorylation caused by tumour heterogeneity [21]. Both patients were injected with relatively low doses of ^68^Ga-PSMA. However, our experiences with this tracer suggest that similar or even lower doses may be suitable to identify lesions with high target to background contrast [26]. As there were no further differences regarding other parameters, it is more probable that PSMA expression in these lesions is low, whereas choline transport and choline kinase activity are high.

Although further analyses are needed to confirm these findings and to define sensitivity and specificity, our data suggest an ability of the presented PSMA ligand to detect low differentiated PC lesions as well (low PSA levels despite multiple metastases and high initial GSC, e.g. patients 12 and 15). On the other hand, evidence exists for a low choline uptake in patients with poorly differentiated PC. In such cases ^18^F-fluorodeoxyglucose (FDG) PET/CT can be used to improve the detection of lesions [30]. In contrast, PSMA expression is usually higher in lesions with higher GSC than in lesions with lower GSC [31]. The presented PSMA ligand, therefore, might be useful to reduce multiple investigations.

Although PSMA-negative PC seems to be rare [21, 32], we cannot exclude the possibility of false-negative PET/CT imaging in patients without pathological findings. One other aspect is the fact that in patients investigated by histology there were no false-negative or false-positive findings indicating a high sensitivity and specificity of the PSMA ligand. This is similar to our experiences with different patients outside this study examined only with the PSMA ligand who were investigated by biopsy (data not yet published). Another aspect is that in cases of multiple metastases or metastases with difficult access, biopsy is neither indicated nor ethical. However, multiple other patients who were treated by selective radiation and ^131^I-PSMA ligands followed by PSA decrease demonstrated sufficiently that the PSMA-positive lesions were indeed PC.

With the exception of sensitivity and specificity, the most significant aspects of our study fulfilled the STARD criteria. However, our study simply followed the experiences of daily routine and therefore might be of relevance until results that conform 100 % with STARD are available in the future.

One additional aspect of the presented ^68^Ga-labelled PSMA ligand is the fact that no (cost-intensive) cyclotron is needed. ^68^Ga can be extracted from a commercially available ^68^Ge/^68^Ga radionuclide generator. In contrast, radiolabelling choline tracers requires isotopes produced by a cyclotron (e.g. ^18^F or ^11^C).

Further analyses are also required to evaluate the characteristics of different types of metastases visible by PSMA PET. Also the question has to be addressed whether dynamic imaging might improve detection of lesions close to the urinary bladder. In addition, we hope that future studies will compare the HBED-CC conjugated PSMA ligand and other promising methods of imaging PC, such as PET imaging with ^18^F-FACBC [14].

### Conclusion

This study presents a retrospective comparison between the established ^18^F-fluoromethylcholine-based PET/CT and a novel method of PET imaging with a ^68^Ga-labelled PSMA ligand in the diagnosis of recurrent PC. Our experience with ^68^Ga-PSMA PET/CT strongly suggests that this is an easy to handle method which can detect PC relapses and metastases with significantly improved contrast when compared to choline-based PET/CT. Nevertheless, the most significant advantages of ^68^Ga-PSMA PET/CT are the sensitive detection of lesions even at low PSA levels, of even small lymph node metastases (primarily due to a high radiotracer uptake) and of central bone and liver metastases due to low background signal.

## Electronic supplementary material

Below is the link to the electronic supplementary material.

Table 2 Comparison of uptake and contrast in ^68^Ga-PSMA PET/CT and ^18^F-fluoromethylcholine PET/CT (PDF 174 kb)

Table 3 Characteristics of different types of metastases (PDF 209 kb)

Table 4 Characteristics of different types of metastases (PDF 82 kb)
