# Supplementary material for: Comparison of PET imaging with a 68Ga-labelled PSMA ligand and 18F-choline-based PET/CT for the diagnosis of recurrent prostate cancer
Source: Eur J Nucl Med Mol Imaging. 2013 Sep 27;41(1):11–20. doi: 10.1007/s00259-013-2525-5 (PMC3843747; doi:10.1007/s00259-013-2525-5)
Supplement: Supplementary file 1 — Comparison of uptake and contrast in 68Ga-PSMA PET/CT and 18F-fluoromethylcholine PET/CT (PDF 174 kb) [file 259_2013_2525_MOESM1_ESM.pdf]

Supplementary Data

Table 2 Comparison of uptake and contrast in ^{68}Ga-PSMA-PET/CT and ^{18}F-fluoromethylcholine-PET/CT

SUV_{max} in tumor suspicious lesions	significantly higher SUV_{max} in ^{68}Ga-PSMA-PET/CT	significantly higher SUV_{max} in ^{18}F-fluorcholine-PET/CT	without significant difference between ^{68}Ga-PSMA-PET/CT and ^{18}F-fluorcholine-PET/CT
Lymph node metastases (n=40)	n=38 (95%)	n=1 (2.5%)	n=1 (2.5%)
Bone metastases (n=23)	n=17 (73.9%)	n=6 (26.1%)	n=0
Local relapses (n=10)	n=6 (60%)	n=2 (20%)	n=2 (20%)
Soft tissue metastases (n=5)	n=2 (40%)	n=3 (60%)	n=0
**Ratio between SUV_{max} of tumor lesions and SUV_{max} of background**	**significantly higher ratio in ^{68}Ga-PSMA-PET/CT**	**significantly higher ratio in ^{18}F-fluorcholine-PET/CT**	**without significant difference between ^{68}Ga-PSMA-PET/CT and ^{18}F-fluorcholine-PET/CT**
Lymph node metastases (n=40)	n=40 (100%)	n=0	n=0
Bone metastases (n=23)	n=21 (91.3%)	n=2 (8.7)	n=0
Local relapses (n=10)	n=9 (90%)	n=0	n=1 (10%)
Soft tissue metastases (n=5)	n=3 (60%)	n=0	n=2 (40%)
