# Supplementary material for: Comparison of PET imaging with a 68Ga-labelled PSMA ligand and 18F-choline-based PET/CT for the diagnosis of recurrent prostate cancer
Source: Eur J Nucl Med Mol Imaging. 2013 Sep 27;41(1):11–20. doi: 10.1007/s00259-013-2525-5 (PMC3843747; doi:10.1007/s00259-013-2525-5)
Supplement: Supplementary file 2 — Characteristics of different types of metastases (PDF 209 kb) [file 259_2013_2525_MOESM2_ESM.pdf]

Table 3 Characteristics of different types of metastases

SUV_{max} in tumor suspicious lesions	average SUV_{max} (± SD) of Choline	average SUV_{max} (± SD) of PSMA	minimum (Choline)	minimum (PSMA)	maximum (Choline)	maximum (PSMA)	median (Choline)	median (PSMA)
Lymph node metastases (n=40)	2.8 (± 2.0)	16.2 (± 18.9)	0.7	2.0	8.6	100.0	2.1	7.7
Bone metastases (n=23)	6.1 (± 2.6)	9.2 (± 3.8)	2.9	2.1	15.5	16.3	5.8	10.6
Local relapses (n=10)	4.3 (± 1.2)	5.4 (± 2.9)	2.6	2.2	5.8	10.5	4.3	3.8
Soft tissue metastases (n=5)	5.2 (± 3.2)	5.4 (± 5.9)	2.6	0.7	11.3	16.9	4.0	3.1
**Ratio between SUV_{max} of tumor lesions and SUV_{max} of background**	**average ratio (± SD) of Choline**	**average ratio (± SD) of PSMA**	**minimum (Choline)**	**minimum (PSMA)**	**maximum (Choline)**	**maximum (PSMA)**	**median (Choline)**	**median (PSMA)**
Lymph node metastases (n=40)	2.6 (± 1.9)	31.5 (± 33.3)	0.6	4.0	7.4	142.9	1.9	17.3
Bone metastases (n=23)	3.3 (± 2.1)	13.6 (± 11.7)	1.1	1.8	8.9	40.8	3.0	8.4
Local relapses (n=10)	4.1 (± 1.6)	11.5 (± 7.5)	2.2	4.4	7.3	26.3	3.7	8.8
Soft tissue metastases (n=5)	3.5 (± 2.4)	8.8 (± 11.3)	0.9	1.0	7.7	31.0	3.6	2.6

Average SUV_{max} of different lesions and their ratio to background including standard deviation, minimum, maximum and median.
