# Supplementary material for: Comparison of PET imaging with a 68Ga-labelled PSMA ligand and 18F-choline-based PET/CT for the diagnosis of recurrent prostate cancer
Source: Eur J Nucl Med Mol Imaging. 2013 Sep 27;41(1):11–20. doi: 10.1007/s00259-013-2525-5 (PMC3843747; doi:10.1007/s00259-013-2525-5)
Supplement: Supplementary file 3 — Characteristics of different types of metastases (PDF 82 kb) [file 259_2013_2525_MOESM3_ESM.pdf]

Table 4 Characteristics of different types of metastases

Patients	initial GSC	Dosage (MBq)	PSA (ng/ml)
**with path. findings in choline-PET/CT (n=26)**	**7.2 (± 1.1)**	**245.3 (± 61.0)**	**14.6 (± 27.9)**
without path. findings in choline-PET/CT (n=11)	8 (± 1.0)	241.9 (± 40.2)	2.8 (± 3.9)
**with path. findings in PSMA-PET/CT (n=32)**	**7.4 (± 1.1)**	**140.6 (± 45.5)**	**12.5 (± 25.6)**
without path. findings in PSMA-PET/CT (n=5)	7.8 (± 1.0)	133.4 (± 50.3)	2.0 (± 0.8)
